# Terrestrial Microorganisms: Cell Factories of Bioactive Molecules with Skin Protecting Applications

**DOI:** 10.3390/molecules24091836

**Published:** 2019-05-13

**Authors:** Laure-Anne Peyrat, Nikolaos Tsafantakis, Katerina Georgousaki, Jamal Ouazzani, Olga Genilloud, Ioannis P. Trougakos, Nikolas Fokialakis

**Affiliations:** 1 Department of Pharmacognosy and Natural Products Chemistry, Faculty of Pharmacy, National and Kapodistrian University of Athens, 15771 Athens, Greece; ntsafantakis@pharm.uoa.gr (N.T.); kat_georgousaki@hotmail.com (K.G.); 2 Institut de Chimie des Substances Naturelles (ICSN), Centre National de la Recherche Scientifique, 91198 Gif-sur-Yvette, France; Jamal.Ouazzani@cnrs.fr; 3 Fundación MEDINA, 18016 Granada, Spain; olga.genilloud@medinaandalucia.es; 4 Department of Cell Biology and Biophysics, Faculty of Biology, National and Kapodistrian University of Athens, 15784 Athens, Greece; itrougakos@biol.uoa.gr

**Keywords:** terrestrial microorganisms, antioxidant, photo-protective, skin-whitening, cosmetics

## Abstract

It is well known that terrestrial environments host an immense microbial biodiversity. Exposed to different types of stress, such as UV radiation, temperature fluctuations, water availability and the inter- / intra-specific competition for resources, terrestrial microorganisms have been evolved to produce a large spectrum of bioactive molecules. Bacteria, archaea, protists, fungi and algae have shown a high potential of producing biomolecules for pharmaceutical or other industrial purposes as they combine a sustainable, relatively low-cost and fast-production process. Herein, we provide an overview of the different bioactive molecules produced by terrestrial microorganisms with skin protecting applications. The high content in polyphenolic and carotenoid compounds produced by several strains, as well as the presence of exopolysaccharides, melanins, indole and pyrrole derivatives, mycosporines, carboxylic acids and other molecules, are discussed in the context of their antioxidant, photo-protective and skin-whitening activity. Relevant biotechnological tools developed for the enhanced production of high added value natural products, as well as the protecting effect of some antioxidant, hydrolytic and degrading enzymes are also discussed. Furthermore, we describe classes of microbial compounds that are used or have the potential to be used as antimicrobials, moisturizers, biosurfactants, pigments, flavorings and fragrances.

## 1. Introduction

Microorganisms are extremely diverse organisms, including bacteria, archaea, protists, fungi and algae. In recent decades, there has been great progress on exploiting the immense chemical diversity available from the abundant microbial world [1,2]. After the discovery of the fungal metabolite penicillin in 1928, which was the beginning of the golden age of microbial-derived natural products and pharmaceuticals, treatments for fungal and parasitic infections as well as for several types of cancers followed [3]. In the forties and early fifties, almost all groups of important antibacterial antibiotics (tetracyclines, cephalosporins, aminoglycosides, macrolides) were discovered, while in the fifties and sixties, antitumor, antiviral and non-antibiotic-enzyme-inhibitory-metabolites were isolated, mainly from *Streptomyces* species [4].

The successful and wide utilization of microbial metabolites in various therapeutic areas (e.g., cyclosporine as immunosuppressant, doxorubicin as anticancer, and statins as cardio protective agents), as well as the wide application in livestock and agriculture (e.g., the antiparasitic avermectin, the feed additive monensin and the herbicide glufosinate) [5] were important features for broadening the research of bioactive microbial products in other sectors. In fact, in the last decade, microorganisms have attracted a great deal of attention as potential leading producers of promising compounds for cosmetic and/or cosmeceutical purposes [6]. Among these compounds, polyphenols, quinones and aldehydes have been reported in several studies as functional active ingredients for the maintenance of skin homeostasis (e.g., antioxidants, UV protecting, skin whitening) as well as coloring, flavoring, stabilizing and antibacterial agents [2].

**Figure 1:**
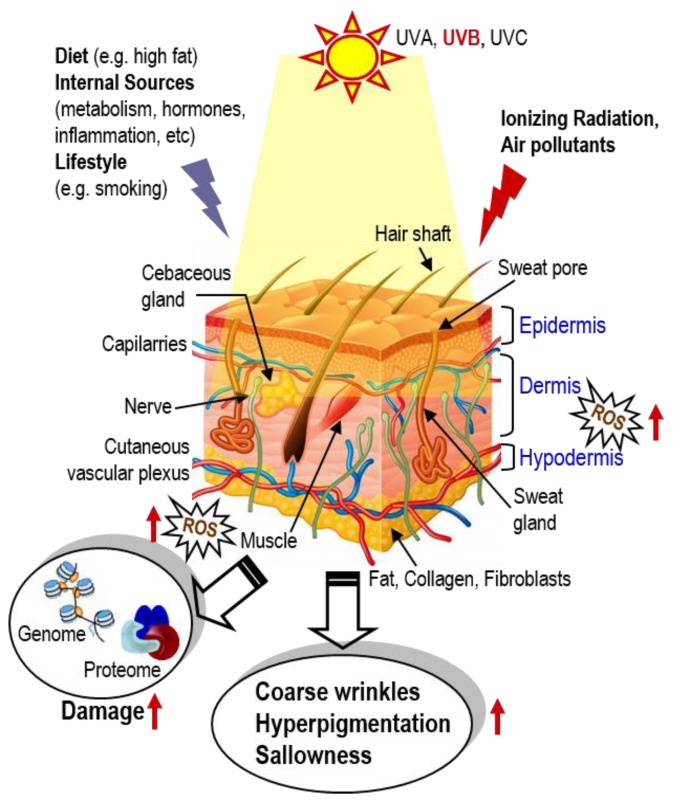
During lifetime, skin is exposed to numerous environmental stressors such as UVA and UVB, ionizing radiation and air pollutants, as well as to stressors that may originate from diet (e.g., high fat diets), internal sources (e.g., metabolism or tissue inflammation) and/or lifestyle (e.g., smoking). While young, these stressors are effectively neutralized by cell protective mechanisms (e.g., the antioxidant transcription factor Nrf2). During aging, defenses are compromised, resulting in accumulating ROS, genome and proteome damage; damage of biomolecules then disrupts normal cell signaling and homeodynamics, resulting in (among others) coarse wrinkles, hyperpigmentation and skin shallowness.

Among the various environmental factors affecting skin homeostasis, ultraviolet (UV) irradiation is the most dangerous component, as it can cross the epidermis and reach the upper dermis. Additional parameters that affect all skin layers and thus contribute to skin aging are dietary (e.g., high fat diet) and lifestyle habits (e.g., smoking), various air pollutants, as well as internal factors such as metabolism, hormones, inflammatory processes, etc. [7,8,9] (Figure 1). Damaging agents modulate numerous molecular events and signaling pathways that (among others) lead to mitochondrial dysfunction, increased genome and proteome damage; increased synthesis and activity of matrix metalloproteases, decreased collagen production, triggering of stress-induced premature senescence (SIPS) and accumulation of the inflammatory senescence-associated secretory phenotype (SASP) [8,9,10,11]. The maintenance of a highly effective intra- or extracellular defense system capable of protecting against the adverse effects of irradiation and other stressors is crucial for safeguarding skin homeostasis. Adverse effects are macroscopically characterized by the loss of skin tone and an increase of wrinkles, dehydration (due to increased epidermal thickness), hyperpigmentation and sallowness (yellowing or pale tinted skin) (Figure 1).

Despite the large number of individual studies and evidences for the potential use of terrestrial microorganisms in the fast-growing cosmetic sector, so far no systematic review has addressed their applications. It is worth mentioning that the global market for cosmetic and cosmeceutical products was valued at USD 532.4 billion in 2017, and is expected to reach a market value of USD 805.6 billion by 2023, registering a CAGR (Compound Annual Growth Rate) of 7.14% during 2018–2023 [12].

In the current study, we provide an overview of the different bioactive compounds with skin protecting effect (and thus of cosmetic and cosmeceutical interest) isolated, from a broad range of terrestrial microorganisms including bacteria, algae, fungi and protists. Examples of biomolecules with skin protecting interest that are heterologously produced and/or biotransformed are included. The term “terrestrial” encompasses microorganisms from soil and freshwater, plant endophytes, and lichens. Marine microorganisms and mushrooms (all Basidiomycota and Ascomycota) are excluded, as they have been recently reviewed [2,13]. Representative bioactive compounds from terrestrial microorganisms with antioxidant, photo-protective, and skin-whitening activity, along with antimicrobial and moisturizing agents, pigments, fragrances and flavors are discussed. A detailed table including bioactive molecules, the source organisms and their habitat, the biological activity, as well as their presence in the list of cosmetic substances and ingredients of the European Union (CosIng inventory [14]) is provided.

## 2. Antioxidants

Oxidative stress is one of the prevailing causes of skin aging due to increased production and/or accumulation of Reactive Oxygen (ROS) and Nitrogen Species (RNS). Imbalance between their production and the endogenous antioxidant defense mechanisms may result in cellular oxidative stress, causing wrinkling, drying, photo-aging, pigmentation and elastosis of the skin. In addition, accumulation of free radicals may be responsible for cutaneous inflammation and skin cancer [15].

Reactive oxygen species (ROS) are formed as either by-products of normal metabolism (e.g., mitochondrial oxidative phosphorylation), as well by NAD(P)H oxidases, or by exogenous sources such as atmospheric pollutants, UV light, X- or gamma-rays [16]; if their concentration exceeds the cellular antioxidant capacity, ROS cause oxidative stress and damage to all cellular biomolecules [10].

Topical antioxidant products could act as scavengers of reactive species, inhibiting the initiation of chain reactions, responsible for cellular oxidative stress [17]. Many reports have demonstrated the ability of marine microorganisms to biosynthesize antioxidant compounds [2]. Concerning terrestrial microbes, compounds with a significant inhibition of oxidation reactions, like polyphenols, carotenoids or exopolysaccharides, are extensively discussed in the following sections.

Bioassays involving the neutralization of different radicals such as the stable radical 2,2-diphenyl-1-picrylhydrazyl (DPPH), the cation radical 2,2′-azino-bis-3-ethylbenzotiazolin-6-sulfonic acid (ABTS), as well as the hydroxyl and nitric oxide radicals are widely applied for determining the in vitro antioxidant potential. Even if the relation to the in vivo antioxidant efficacy was not clearly described, the measured antioxidant activity can give an estimation of the amount of the compounds that can be oxidized under conditions of the assays [18].

### 2.1. Phenolic Compounds

**Figure 2:**
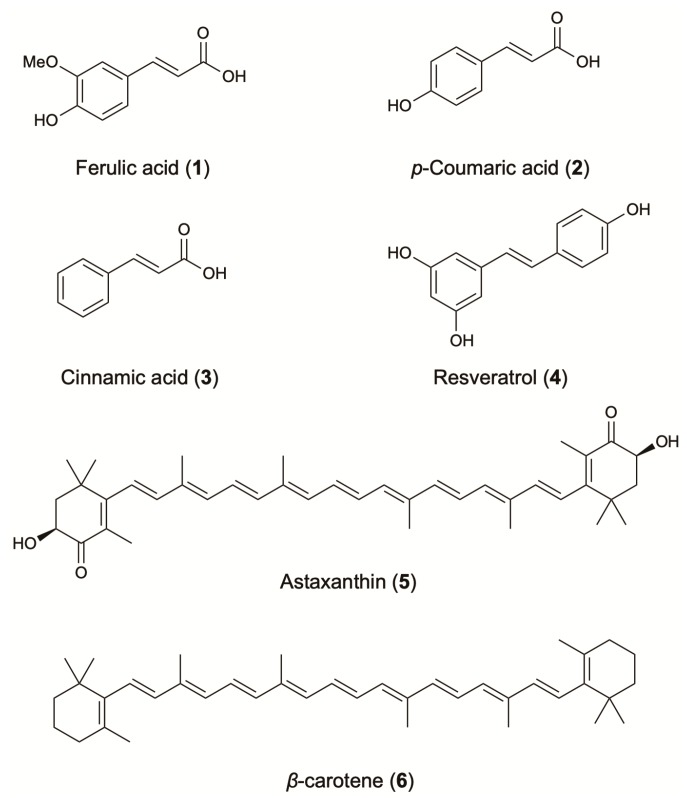
Antioxidant agents from terrestrial microorganisms.

Phenolic compounds are well known for their strong antioxidant and radical scavenging activity, as well as for their interaction with different pharmacological targets. The strong correlation between the microbial phenolic content and the antioxidant activity has been shown by several authors using different microorganisms. Huang et al. confirmed this positive correlation during their investigation of fungal endophytes isolated from medicinal Chinese plants [19]. The strong contribution to the antioxidant activity was also confirmed in *Aspergillus austroafricanus*, an endophytic fungus isolated from *Zingiber officinale* rhizome. HPLC analysis of the crude extract showed mainly the presence of hydroxycinnamic acids such as ferulic acid (**1**), *p*-coumaric acid (**2**) and cinnamic acid (**3**) [20] (Figure 2). Those molecules are well known in the plant kingdom and have been extensively studied for their antioxidant capacity (Table 1).

Similar conclusions were drawn using cultures of the microalgae *Arthrospira platensis* [21] and of other *Arthrospira* sp. [22,23]. Simple phenolics and hydroxycinnamic acids, such as gallic acid, chlorogenic acid, ferulic acid, and caffeic acid have been isolated from different species of microalgae e.g *Chlorella vulgaris, Haematococcus pluvialis, Diacronema lutheri, Phaeodactylum tricornutum, Tetraselmis suecica, Ankistrodesmus* sp., *Spirogyra* sp., *Euglena cantabrica, Caespitella pascheri*, and *Porphyridium purpureum* [24,25,26].

Studies on terrestrial cyanobacterial species from the genera Anabaena, Nostoc, Nodularia, Microcheate, Oscillatoria, Synechocystis, Hapalosiphon, Mastigocladus, Scytonema, Westiellopsis, Cylindrospermum, Aulosira, Chroococcus, Lyngbya, Calothrix, Dichothrix, Phormidiochaete, Limnothrix and Phormidium have also reported the correlation of their antioxidant activity with their total phenolic content. Chlorogenic and gallic acid were identified as main phenolics in several cyanobacterial species, with Dichothrix sp. being one the most efficient producer of those compounds (77.9 µg/g and 24.4 µg/g fresh weight, respectively) [26].

Resveratrol (**4**), another well-known natural compound produced by plants, has recently been reported from endophytes isolated from grapevine varieties [27,28]. It is considered one of the most famous compounds for its unique anti-aging properties (Figure 2). It has been widely reported to be a strong inhibitor of ROS production and protein oxidation and a more effective agent than vitamins E and C against lipid peroxidation [29]. Microorganisms have been successfully considered for the production of resveratrol, since its synthesis and/or its extraction from plants is considered inefficient due to high requirements of organic solvents, biomass and low final yield. Resveratrol was first industrially produced in 2009, using *Saccharomyces cerevisiae*. Since this development, different methods such as bioconversion and genetic engineering have been used in order to obtain higher yields. For instance, resveratrol has been produced by *Alternaria* sp. (1.4 μg/L), and by genetically modified *S. cerevisiae* (531.4 mg/L), and *E. coli* (2370 mg/L) [30]. The molecules discussed, as well as additional microbial phenolic compounds that have antioxidant or other related biological activities, are presented in Table 1.

### 2.2. Carotenoids

Carotenoids are the most common natural pigments; they are well known for their powerful antioxidant activity, as they are very efficient physical quenchers of singlet oxygen and scavengers of other ROS. Carotenoids are also well known for their capacity to act as quenchers of photosensitization products, giving them photo-protective properties [109].

In the last decade, the interest in microbial fermentation for the production of natural carotenoids has increased. Carotenoid production by bacteria, asporogenous yeasts, filamentous fungi [110] and microalgae [111] has been extensively reported, with cyanobacteria to be the most prominent source [112]. Accordingly, carotenogenic microbes *Xanthophyllomyces dendrorhous*, *Blakeslea trispora*, and *Haematococcus pluvialis* have been widely used in large-scale processes. Furthermore, the transformation of the non-carotenogenic microbes *E. coli*, *S. cerevisiae*, *Candida utilis*, and *Zymomonas mobilis*, with carotenoid genes from selected microbes has been successfully applied for the production of carotenoids [113]. In fact, *E. coli* in fed-batch fermentation produced 72.6 mg/g cdw (cell dry weight) of *β*-carotene [1] and 1.44 g/L of lycopene [49], while astaxanthin production was enhanced 1.4-fold compared to the *X. dendrorhous* parental strain, reaching 1.25 mg/L (Table 1) [46]. Astaxanthin (**5**), *β*-carotene (**6**) and lutein are the carotenoids with the highest added value (Figure 2) [114]. The oxycarotenoid lutein is mainly produced by microalgae of the genus *Chlorella*, *Dunaliella*, and *Haematococcus* [114]. Its profound effect on the antioxidant defense system is attributed to its chemical structure. In *in vitro* systems, it significantly scavenged the superoxide (IC_50_: 21 μg/mL), the hydroxyl (IC_50_: 1.75 μg/mL), the nitric oxide (IC_50_: 3.8 μg/mL), and the DPPH (IC_50_: 35 μg/mL) radical and inhibited lipid peroxidation (2.2 μg/mL). In in vivo systems, it has been proved to be an effective scavenger of superoxide radical (IC_50_: 21 μg/mL) [51].

### 2.3. Exopolysaccharides (EPSs)

EPSs are high-molecular-weight carbohydrate polymers demonstrating strong scavenging activities, metal chelating ability and lipid peroxidation inhibition. These compounds are among the most exploited bioactive substances for their anti-aging capacity [115].

EPSs are mainly biosynthesized by bacteria and fungi. The ability of a microorganism to produce antioxidant EPSs was first introduced with the study of *Paenibacillus polymyxa*. This endophytic bacterium, isolated from the root of *Stemona japonica*, produces different EPSs with strong scavenging activity against the superoxide and the hydroxyl radical [116,117] (Table 1). When tested at a concentration of 1 mg/mL, the scavenging effect of the crude EPS against the superoxide radical was 74.38%, while the activity of the purified EPS-1 and EPS-2 was higher than ascorbic acid. At the same concentration, EPS, EPS-1, EPS-2 were also very effective against the hydroxyl radical [56]. EPS-1 and EPS-2 were composed of mannose, fructose and glucose in molar ratios of 2.6:29.8:1 and 4.2:36.6:1, respectively. Since this discovery, many endophytes were found to produce antioxidant EPSs. A characteristic case is the purified rhamno-galactan fraction of *Fusarium solani* and *Bacillus cereus* isolated from *Alstonia scholaris* and *Artemisia annua L*., respectively. This EPS fraction showed a significant scavenging activity against the DPPH, (IC_50_:0.6 mg/mL), the superoxide (IC_50_: 2.6 mg/mL) and the hydroxyl radical (IC_50_: 3.1 mg/mL) [54,55].

Optimization of cultivation parameters of *P. polymyxa* using sucrose, yeast extract and CaCl_2_ showed an EPSs yield of 35.26 g/L (18.74%), which was 1.55-fold higher compared to the original medium [57]. EPSs structures are in a great variety. EPSs isolated from the culture medium of the endophytic fungus *Aspergillus* sp. were mainly composed of mannose and galactose (89.4:10.6) [59], while EPSs isolated from the endophytic bacteria *Burkholderia tropica* were mainly composed of rhamnose, glucose and glucuronic acid (2:2:1) [60]. Antioxidant EPSs have also been isolated from the terrestrial microalgae *Rhodella reticulata*. Its extracellular polysaccharides showed strong antioxidant activity, significantly higher than α-tocopherol. The radical scavenging ability against the superoxide radical of the deproteinized extracellular polysaccharide reached 328.48 U/L, compared to 174.03 U/L of α-tocopherol [118].

### 2.4. Enzymes

Enzymes are produced by microorganisms as a primary cell protective detoxification mechanism (e.g., from ROS) as they catalyze the removal of ROS through the formation of less reactive molecules such as oxygen or water. Superoxide dismutases, catalases, and peroxidases are involved in these mechanisms.

Superoxide dismutases (SODs) catalyze the neutralization of two superoxide radicals by the addition of two hydrogen ions to form hydrogen peroxide and oxygen. Belonging to the family of metalloisozymes, SODs are differentiated in their metal cofactor: Ni-SOD, CuZn-SOD, Fe-SOD and Mn-SOD; the last three are commonly found in microalgae. SOD biosynthesis is directly correlated with level of cellular ROS. In fact, a study carried out on microalgae *Scenedesmus vacuolatus* and *Pinnularia viridis* showed that the concentration and SOD activity is correlated with ROS related stress [119,120]. Similarly, the elimination of ROS in most *Streptococcus* and *Lactococcus* bacterial spp., conforms to this general antioxidant defense system since both genera express MnSOD. However, these bacteria possess only one type of SOD, namely the Mn-containing enzyme (MnSOD), rendering this enzyme an essential part of the antioxidant cell machinery [121].

Catalases contain porphyrin heme active sites that degrade hydrogen peroxide into water and oxygen [119]. One molecule of catalase is able to convert six billion molecules of hydrogen peroxide each minute [122]. In the yeast *S. cerevisiae*, the overexpression of catalase reduces lactic acid-induced oxidative stress [123]. Furthermore, a study involving the single-cell green alga *Chlamydomonas reinhardtii* showed that hydrogen peroxide from the media was faster degraded when the catalase inhibitor aminotriazole was absent; thus, catalase is one of the major enzymes involved in ROS detoxification [124].

Finally, peroxidases catalyze the oxidation of several substrates by hydrogen peroxide. Ascorbate, cytochrome C, pyrogallol, and glutathione are examples of these substrates. As for the other antioxidant enzymes, the induction of peroxidases activity upon ROS accumulation seem to be concentration- and time-dependent [119].

## 3. Photo–Protective Agents

Ultraviolet A (UVA, 315–400 nm) and ultraviolet B (UVB, 280–315 nm) play a major role in skin cell damage. UVA is mainly involved in the creation of ROS while UVB heavily affects DNA and proteins integrity. To protect themselves against UV radiation, terrestrial microorganisms have developed several strategies, one of which is the accumulation of photo-protective compounds [2].

Despite the evidence that several compounds from microorganisms have photo-protective activities, there has been surprisingly little work carried out involving in vivo skin models. This might be partially explained by the fact that the EU has banned the in vivo testing of cosmetics since 2013. Thus, potential skin protecting effects have been established based on existing in vitro studies [125].

### 3.1. Melanins

Bacteria, fungi and protists are able to produce a diverse group of pigments. Melanized fungi are mostly black yeasts, and melanized bacteria belong mainly to Actinobacteria [126]. 

The basic role of melanins in microorganisms are still a matter of controversy and speculation. The fact that these compounds are interceptors of UV photons leads to a lower vulnerability of micro-ecosystems to UV radiation. Melanins are also involved in energy production due to their ability to accept electrons. Finally, in some pathogenic microorganisms, these compounds act like virulence factors, lowering the defense mechanisms of the host [127].

The term melanin encompasses three polymeric substances; eumelanins, pheomelanins and allomelanins. Bacteria contain mostly eumelanins and allomelanins, whereas fungi mostly express allomelanins [126]. Fungal melanins have been isolated from *Cryptococcus neoformans*, *Candida albicans*, *Aspergillus* sp., *Sporothrix schenckii*, *Fonsecaea pedrosoi*, *Paracoccidioides brasiliensis*, *Coccidioides* sp., and *Histoplasma capsulatum* [128]. Melanins are also widespread in a variety of bacteria, like *E. coli*, *B. cereus*, *Klebsiella* sp., *Pseudomonas aeruginosa*, *Pseudomonas stutzeri*, *Bacillus thuringiensis*, *Vibrio cholera* and *Streptomyces kathirae* [129]; the last has been selected as an ideal microorganism for melanin production. Under optimal conditions, the yield was maximized at 13.7 g/L. In that study *S. kathirae* was identified as an excellent candidate for industrial-scale production of melanins [67].

### 3.2. Indole and Pyrrole Derivatives

**Figure 3:**
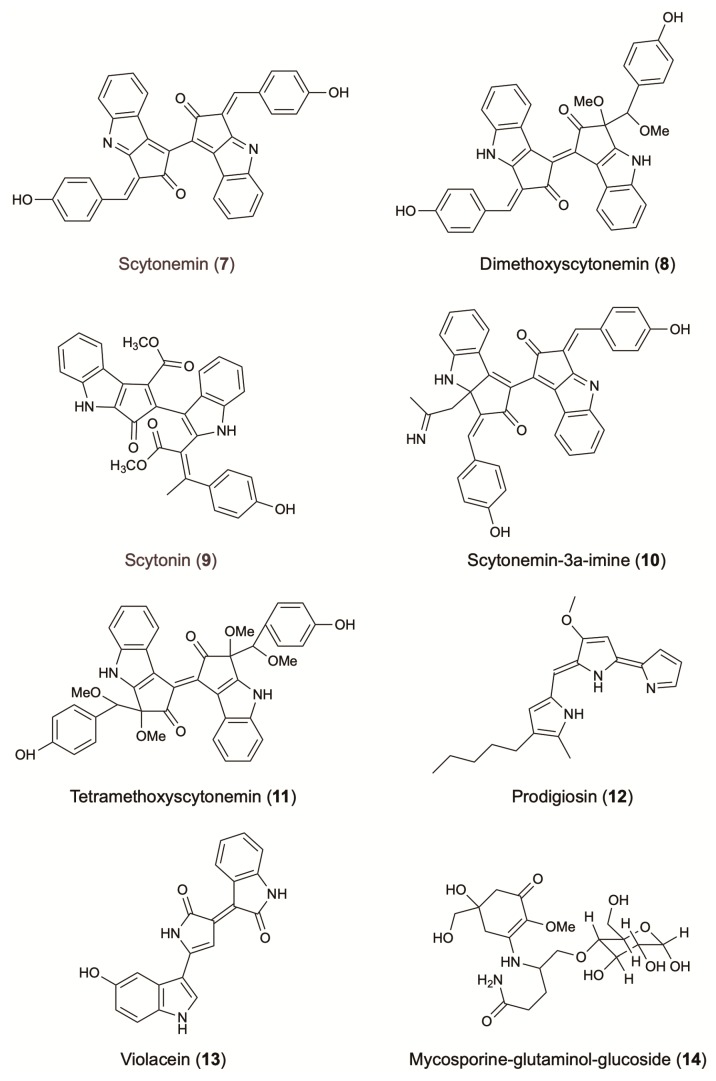
Photo-protective agents from terrestrial microorganisms.

Scytonemin (**7**) is a yellow to brown alkaloid pigment composed of an indolic and a phenolic subunit. Until now, only four different derivatives have been reported: dimethoxyscytonemin (**8**), scytonin (**9**), scytonemin-3a-imine (**10**) and tetramethoxyscytonemin (**11**) (Figure 3). Known for their strong UV-absorbing function and free radical scavenging capacity, scytonemin and its derivatives are excellent candidates for skin protecting purposes. Scytonemin prevents up to 90% of solar UV radiations from entering the cell. The strong radical scavenging activity of this compound (IC_50_: 36 µM against the ABTS radical), combined with its localization in the bacterial cell wall explains its protective role and the inability of UV-A radiation to cross the cellular envelope [130,131].

Almost exclusively synthesized by cyanobacteria from extreme environments, scytonemin (**7**) has been described in more than 300 cyanobacterial species, many of them terrestrial; e.g., *Nostoc commune*, *Nostoc microscopicum*, *Phormidium* sp. and *Pleurocapsa* sp. Scytonemin is also found in *Scytonema hoffmani* together with dimethoxyscytonemin (**8**), tetramethoxyscytonemin (**11**) and scytonin (**9**) [132]. To induce scytonemin (**7**) biosynthesis, modulation of temperature or photo-oxidative stress has to be combined with osmotic stress and periodic desiccation [126]. For industrial applications, the production of the UV-protecting scytonemin has been optimized in *N. commune* to yield 758 µg/g [73] (Table 1).

Prodigiosin (**12**) is characterized by a common pyrrolyl dipyrromethene skeleton containing a 4-methoxy-2,2′-bipyrrole ring system (Figure 3). This red pigment is mainly produced by strains belonging to the bacterial genus *Serratia* [75]. Well known for its antimalarial, antibacterial, and anticancer activity, prodigiosin has also demonstrated UV protective activity. When used as an additive in commercial sunscreens (4% w/w prodigiosin), the sunscreen protection factors (SPF) increased by 20–65%. In the same study, addition of 4% (*w*/*w*) of prodigiosin in photo-protective leaf extracts of *Aloe vera* and *Cucumis sativus* fruits showed an increasing of SPFs up to 3.5 orders of magnitude [133]. Bacteria *Pseudomonas magneslorubra*, *Vibrio psychroerythrous*, *Vibrio gazogenes*, *Alteromonas rubra*, and *Rugamonas rubra*, along with actinomycetes, such as *Streptomyces rubrireticuli* and *S. longisporus ruber*, have been studied for their capacity to produce prodigiosin or its derivatives [133]. Improvement in the production of prodigiosin (277 mg/L) was reported by the addition of a ram horn peptone (RHP, 0.4% *w*/*v*) in the culture media of *S. marcescens* MO-1 [75] (Table 1).

Violacein (**13**) is a purple pigment that presents an unusual structure consisting of a 2-pyrollidone and an oxindole ring system connected by a double bond, and a 5-hydroxyindole unit (Figure 3) [134]. Known to possess antibacterial effects against *Staphylococcus aureus* and other Gram-positive pathogens, violacein can also act as a photo-protective agent against UV irradiation. This compound absorbs at visible wavelengths and presents a broad absorption band extended out to 700 nm [69]. When used as an additive in commercial sunscreens (4% *w*/*w* violacein), the SPFs increased by 10–22%. Furthermore, the addition at 4% (*w*/*w*) of violacein in photo-protective extracts of *A. vera* leaves and *C. sativus fruits*, showed an increasing of SPFs up to 3.5 orders of magnitude [133]. Violacein is mainly produced by the bacterial strains *Janthinobacterium lividum*, *Pseudoalteromonas sp*. and *Chromobacterium violaceum* (Table 1). It is worth mentioning that the medium pH, culture volume, concentration of potassium nitrate, and L-tryptophan, affect significantly violacein production. In fact, the cultivation of *C. violaceum*, isolated from various plant waste sources, in a medium supplemented with sugar bagasse and L-tryptophan 10% (*v*/*v*), increased the final yield production of violacein to 0.82 g/L [70]. Similarly, optimized cultivation parameters of *Duganella* sp. increased by 4.8 folds the final yield of crude violacein (1.62 g/L) [71].

### 3.3. Mycosporines and Mycosporine-Like Amino Acids (MAAs)

Originally detected in the mycelia of terrestrial basidiomycetes, mycosporines present a central cyclohexenone or cyclohexenimine ring and a wide variety of substitutions. Mycosporine-like amino acids are imine derivate of mycosporines. The ring absorbs UV light and dissipates energy as heat, without generating ROS. Cyanobacteria and microalgae can synthesize mycosporines and MAAs, while fungi produce only mycosporines [126] (Table 1).

Mainly known for their photo-protective activity, MAAs are also efficient antioxidants and scavengers of ROS. These activities have led to several patents in the research of natural UV filters [135].

As in other cases, the production of microbial MAAs can be optimized following modification of culturing parameters. Khosravi et al. showed that the combination of UV irradiation and elevated salinity significantly increase the bioaccumulation of MAAs [136]. Indeed, the exposure of terrestrial fungi to UV radiation, desiccation and nutrient scarcity significantly increased the production of the UV-absorbing compound mycosporine-glutaminol-glucoside (**14**) (Figure 3) [137].

## 4. Skin-Whitening Agents

Skin-whitening agents are commercially available for cosmetic and clinical purposes, to obtain lighter skin complexion and treat hyperpigmentary disorders [138]. Uneven pigmentation of the skin may lead to blotches, patches of brown to grey discoloration or freckling which may require cosmetic interventions [13]. Whitening agents act at various levels of melanin production of the skin, either by inhibiting the activity of tyrosinase, the key enzyme in melanogenesis in plants and animals, or by inhibiting the transport of melanosomes from melanocytes to surrounding keratinocytes [139,140,141].

### 4.1. Pyrones

**Figure 4:**
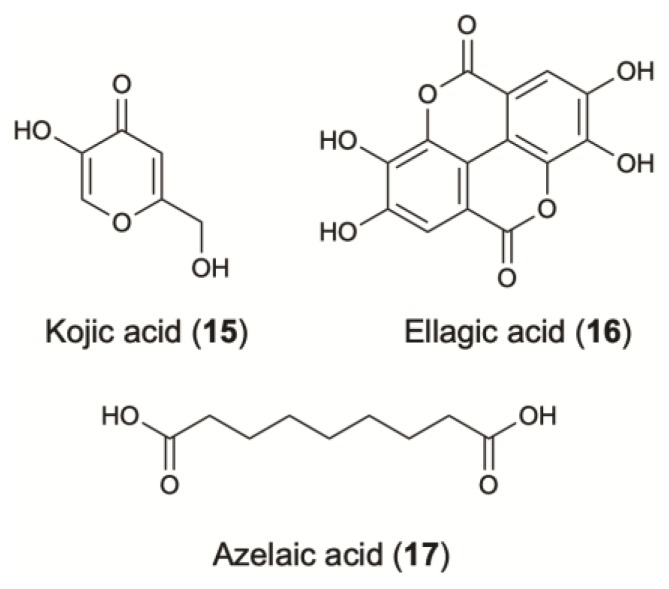
Skin-whitening agents from terrestrial microorganisms.

Kojic acid (**15**) is an inexpensive water-soluble fungal secondary metabolite (Figure 4). It has two OH- groups, the primary at C-7 and the secondary at C-5, which is essential to the radical scavenging and tyrosinase interference activity (IC_50_: 14 µM) [142,143]. The skin depigmenting activity of kojic acid results from the inhibition of the creolase and catecholase activities of tyrosinase. It prevents the conversion of the *O*-quinone to DL-DOPA and dopamine to its corresponding melanin. Decreased melanin content is demonstrated in melanocytes after their treatment with kojic acid [143]. This compound has been extensively used for skin depigmentation (and consequently as a cosmetic agent) with an excellent whitening effect, due to its ability to inhibit tyrosinase activity. 

Mainly produced by *Penicillium* sp. and *Acetobacter* sp., kojic acid has also been isolated from other terrestrial microorganisms, such as *Aspergillus flavus*, an endophytic fungus of *Vigna unguiculata* [81]. To produce this compound, fermentation of *Aspergillus* sp., is widely used. Other strains are also commonly employed, such as *A. oryzae* (0.26 g kojic acid/g glucose), *A. parasiticus* (0.089 g/g glucose) and *A. candidus* (0.3 g/g sucrose). A high yield of 0.453 g/g glucose was obtained with the culture of *A. flavus* [82,83,144] (Table 1).

### 4.2. Phenolic Lactones 

Ellagic acid (**16**) is an antioxidant polyphenol that has generated commercial interest due to recommendations for topical use as a skin-whitening agent (Figure 4). This compound inhibits melanogenesis via the chemical reduction of *O*-quinones (*O*-dopaquinone) and semiquinones [145].

Ellagic acid can be produced from plant tannins via fermentation using different *A. niger* strains [146,147]. A yield of 6.3 and 4.6 mg of ellagic acid/g of dried pomegranate husk were obtained by converting of pomegranate ellagitannins into ellagic acid in a solid state fermentation [85] (Table 1).

### 4.3. Carboxylic Acids

Azelaic acid (**17**) is a saturated dicarboxylic acid which is produced by *Malassezia furfur* (also known as *Pityrosporum ovale*), a yeast that lives on normal skin [91] (Figure 4) (Table 1). It is effective in treating several skin conditions, such as acne, inflammation and hyperpigmentation. As a competitive inhibitor of tyrosinase in vitro, it has been used to treat melasma, Lentigo maligna and post-inflammatory hyperpigmentation. The minimum concentration at which azelaic acid demonstrates its anti-enzymatic activity is 10^−3^ mol/L and it is approximately equal to the 20% content of azelaic acid in a cream applied topically [148,149]. Furthermore, the efficacy of 20% azelaic acid cream is superior than a 2% hydroquinone (HQ) cream while severe side effects were not reported [90,150]. Clinical trials demonstrated that this cream was also effective against melasma when used in parallel with a broad-spectrum sunscreen. Thus, the ability of azelaic acid to reduce the amount of melanin in a specific region of skin tissue as well as the lack of side effects makes it widely used in cosmetic formulations.

Lactic acid is also used as skin whitener (Table 1). At a dose of 500 µg/mL it inhibits melanin formation in a dose-dependent manner without affecting cell growth [151]. Recent studies have shown that species of *Rhizopus* could offer a valuable alternative source for lactic acid production [152]. The filamentous fungus *R. oryzae* converts both glucose and xylose under aerobic conditions into l(+)-lactic acid with yields varying between 0.55 and 0.8 g/g [87].

Poly γ-glutamic acid (γ-PGA) is a natural polymer produced by different species of *Bacillus* (yields vary from 10 to 50 g/L depending on the species) [88] (Table 1). Studies related to the inhibitory effect against mushroom tyrosinase and tyrosinase in B16 melanoma cells reported a dose dependent activity. γ-PGAs, and especially the low molecular weight polymers, has attracted much attention owing to its great potential in cosmetics as skin-whitening agents [153].

### 4.4. Enzymes and Derived Products

The possibility of using melanolytic enzymes in skin lightening was examined by screening the potential melanolytic activity of wild fungal isolates. Among them, *Sporotrichum pruinosum* was the most promising from the very limited number of fungi that decolorize synthetic melanin [154]. As described in the US 20030077236 Patent, compositions containing melanin-degrading enzymes derived from *Aspergillus fumigatus* or *S. cerevisiae* were twice as effective as kojic acid in producing a whitening effect on the skin.

A large variety of compounds with potential skin protecting applications can be obtained through biotechnological processes by using enzymes isolated from terrestrial microorganisms. This is the case of retinol, the most active form of Vitamin A, a skin-whitening agent that has been synthesized by the esterification of palmitic acid using a modified lipase B from *Candida antarctica* (CALB) and a modified lipase from *Pseudomonas fluorescens*, in order to maximize its solubility in water and minimize skin irritation. Other Vitamin A modifications include the esterification with oleic, lactic, succinic or methylsuccinic, catalyzed by CALB or by *Rhizomucor miehei* lipase [155].

A better dermal absorption and a 10% higher skin-whitening activity, as compared to the well-known tyrosinase inhibitor arbutin, was demonstrated by its derivative arbutin undecylenic acid ester which has been enzymatically synthesized using an alkaline protease from *Bacillus subtilis* [94,155]. In addition, α-arbutin glycosides were synthesized by the trans glycosylation reaction of cyclomaltodextrin glucanotransferase from *Bacillus macerans*. Synthesized glucosides exhibited higher inhibition on human tyrosinase than α-arbutin [156].

## 5. Additives and Other Active Ingredients

Additive products provide long-term physical stability, inhibit germination and influence the sensory perception. Recently, the cosmetic industry has been strongly criticized for the addition of chemicals such as formaldehyde, dioxane, parabens, and phthalates. Controversies regarding the human health impact of those synthetic molecules and their analogues has encouraged the research of new additives from natural sources.

### 5.1. Antimicrobial Agents

**Figure 5:**
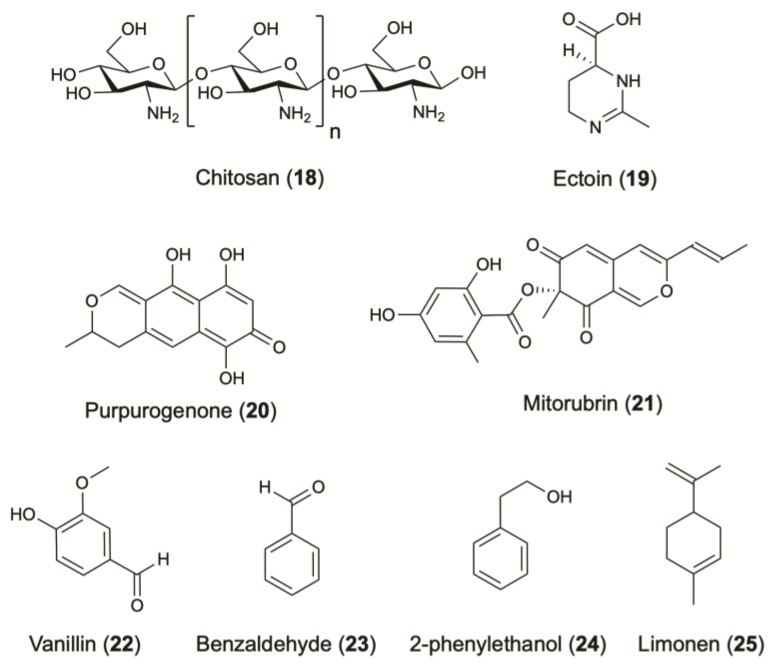
Additives and other active ingredients from terrestrial microorganisms.

One of the most widely used antimicrobial agents against bacteria, viruses and fungi contamination in cosmetics is chitosan (**18**) (Figure 5). This polysaccharide is composed mostly of glucosamine and a variable number of N-acetylglucosamine residues. Although chitosan is present in large amounts in the exoskeleton of crustaceans, insects, crabs, and shrimps, its production is limited due to factors such as seasonality, production sustainability and processing cost. To face these difficulties, chitosan can be produced by an alternative and more effective sources of microbial origin since 22–44% of the cell wall of fungi is composed of chitosan [2]. An optimal production was found in *Rhizopus* *oryzae* (0.5 g/L), *R. japonicus* (0.6 g/L) and *Mucor indicus* (0.75 g/L) (Table 1) [63], while *A. niger*, isolated from the lichen *Roccella montagnei*, showed a higher yield of 1.3 g/L, which was further increased to 1.93 g/L when glucose was added [65]. In addition to the antimicrobial activity, chitosan is known for its emulsifying and delivering properties. This compound has a better water-binding capacity than methyl-cellulose, which is commonly used in cosmetics [2]. Consequently, chitosan and its derivatives, like the copolymer chitin-glucan, can present potential candidates for cosmetic and cosmeceutical formulations. Other examples with anti-aging activity that also combine antimicrobial activity are presented in Table 1.

### 5.2. Moisturizers and Biosurfactants

Concerning moisturizing care, ectoine (**19**) is commonly used for its strong hydration properties (Figure 5). This cyclic amino acid is produced by several bacterial species in response to osmotic stress. *Corynebacterium glutamicum* is widely studied as a microbial cell factory for the biotechnological production of ectoine. The optimization of some cultivation parameters led to the production of 6.7 g/L/day of ectoine [98] (Table 1).

Glycolipids represent an important class of biosurfactants. Among them, sophorolipids and trehalolipids are efficient biosurfactants. Sophorolipids are mainly produced by yeasts belonging to the genus *Candida* (formerly called *Torulopsis*), like *C.* *bombicola, C. petrophilum* and *C. apicola*, while trehalolipids by *Rhodococcus* sp., *Mycobacterium* sp., *Nocardia* sp., and *Corynebacterium* sp. Trehalolipids represent structures with a variation in the number of carbon atoms and the degree of unsaturation.

Rhamnolipids are commonly used in cosmetics as moisturizers and biosurfactants [108]. Rhamnolipids, primarily crystalline acids, are composed of a *β*-hydroxy fatty acid attached by the carboxyl end to a rhamnose sugar molecule and are classified as mono and di-rhamnolipids [157]. Compared to chemical surfactants, biosurfactants have several advantages, because of their better compatibility, lower toxicity and higher biodegradability [158]. Rhamnolipids are mainly produced by *Pseudomonas aeruginosa* as well as by other *Pseudomonas* sp. They are also used in the pharmaceutical industry for their antiviral and antimicrobial properties [159,160] and for others targets related to skin regeneration such as wound healing with reduced fibrosis, cure of burn shock and treatment of wrinkles [161].

### 5.3. Pigments

Microorganisms produce several compounds that can be used as natural pigments. A lot of synthetic dyes have been commercialized, but few of them are eligible in cosmetics. Natural pigments are more stable and less allergenic compared to synthetics [162]. Pigments commonly biosynthesized by fungi include aromatic polyketides such as quinones, anthraquinones, naphthoquinones, melanins, flavins and ankaflavins. Purpurogenone (**20**) and mitorubrin (**21**) are two characteristic examples, produced by the fungus *Penicillium purpurogenum* [95] (Figure 5) (Table 1). Recently, the potential use of terrestrial fungi as a source of natural pigments has been considerably investigated [163,164,165].

Cyanobacteria are an interesting source of pigments, that have the ability to produce phycobiliproteins, which are brilliantly colored fluorescent proteins. Among phycobiliproteins, phycocyanins are already used in diagnostic assays such as flow cytometry, fluorescence activated cell sorting, histochemistry, etc. Their intense blue color allows their use in cosmetics as natural dyes [166]. Phycocyanins are mainly produced by the photoautotrophic cyanobacterium *Arthrospira* *platensis* (3.2 g/L) [167]. However, the unicellular rhodophyte *Galdieria sulphuraria* showed excellent results; this red alga, growing usually in acidic springs, produced c-phycocyanin with a yield of 2.9 g/L [168] (Table 1).

### 5.4. Flavoring and Fragrances

Many flavoring and fragrance compounds on the market are still produced through plant and animal sources. However, a rapid and sustainable alternative is given as such high value compounds can be also produced by microorganisms [169]. Numerous yeasts and terrestrial fungal and bacterial strains are able to synthesize potentially valuable fragrance compounds, including alcohols, aldehydes, esters, fatty acids, ketones, lactones, aromatic compounds and pyrazines [170]. In support, several articles and reviews have been published and offer sufficient information regarding the use of microbial cultures or enzyme preparations for the production of flavor compounds valuable for the cosmetic industry [171,172,173,174]. Vanillin (**22**) is a very good example of a natural fragrance where the increasing demand and value have led to the development of alternative strategies for its production [175] (Figure 5). Strains including *Pseudomonas putida*, *Aspergillus niger*, *Corynebacterium glutamicum*, *Corynebacterium* sp., *Arthrobacter globiformis* and *Serratia marcescens* were successfully introduced for its production by converting eugenol or isoeugenol to vanillin [170].

Benzaldehyde (**23**) is among the most commonly used flavoring agent, with a strong cherry and almond-like aroma. An *E. coli* strain was successfully engineered to produce this aromatic [100,176], while the fungus *Ashbya gossypii* has been tested for its ability to synthetize the rose flavour 2-phenylethanol (**24**) [104]. Among terpenes, limonene (**25**) is one of the most widely used terpene due to its unique citrus scent [169]. Optimization of the expression pathway in *E. coli* led to a yield of 435 mg/L with 1% of glucose as carbon source [177]. When the impact of a different carbon source have been explored, the fermentation using glycerol led to the titers of 2.7 g/L [106] (Figure 5) (Table 1).

## 6. Other Targets of Skin Protecting Interest

Elastase and collagenase inhibitors of microbial origin are promising cosmeceutical agents that worth to be further explored. Elastase, a member of the chymotrypsin family of serine proteases, is responsible primarily for the breakdown of elastin, which is an important protein found within the extracellular matrix of the skin, whose damage has a significant impact in skin ageing. Nostopeptins A and B isolated from the freshwater cyanobacterium *Nostoc minutum* are the only reported inhibitors of elastase (IC_50_: 1.3 and 11.0 µg/mL) [178]. On the other hand, collagen, the major constituent of the skin (80% of skin dry weight), is responsible for the tensile strength. The metalloproteinases named collagenases are capable of cleaving collagen and elastin. To the best of our knowledge, terrestrial microorganisms, apart from the aforementioned example of nostopeptins A and B, have not been investigated thoroughly yet for their ability to produce metabolites with elastase and collagenase inhibitory effects although that large screening programs on terrestrial microorganism and endophytes have been recently presented [179,180].

## 7. Targets for Future Developments

Beyond the above applications of microbial-derived natural products, it is worth mentioning some new cosmeceutical targets with great potential for future development.

It is well known that skin retains its young-looking appearance for many years due to numerous cell genome and proteome protective mechanisms; these are mostly driven by protein machines that execute both DNA and proteome damage responses. Proteome quality control is carried out through the curating activity of the proteostasis network (PN) and is critical for cellular functionality [7,10,11]. Key components of the PN are the two main degradation machineries, namely the autophagy-lysosome and the ubiquitin-proteasome pathways; several short-lived transcription factors are also considered to be part of the PN as they mobilize genomic cytoprotective responses [11]. These, among many others, include Nrf2, which responds to oxidative, electrophilic, and/or proteotoxic stress [11,181,182]. Deregulation of the PN functionality is associated with ageing and it is considered a major risk factor for a wide spectrum of age-related protein conformational diseases such as immunological and metabolic disorders, cardiovascular and neurodegenerative diseases and cancer [11,183]. On the other hand, several studies have shown that the activation of proteostatic modules by genetic, dietary and/or pharmacological interventions increases organismal health- and/or life-span and delays cellular senescence [7,182].

Concomitantly, natural compounds that activate the PN have also been reported to possess anti-aging properties at either cell-based or in vivo models [7,11,182]; likewise, natural products significantly delay the appearance of the aged skin hallmarks. To the best of our knowledge, only few molecules of microbial origin were reported to activate proteostatic modules. Betulinic acid was recently isolated from the endophytic fungi *Phomopsis* sp. and its preferentially activating the chymotrypsin-like proteasomal activity with no or minimal effects on trypsin-like and caspase-like activities [184,185]. The second case of a microbial natural product, that is well known for its anti-aging proprieties is rapamycin. This molecule isolated from *Streptomyces hygroscopicus*, delay cellular senescence through (among others) the inhibition of the TOR pathway and the downstream induced alterations to both autophagy and the rate of protein synthesis [7].

## 8. Conclusions

Naturally derived molecules are traditionally used in skin protection products (Table 1, CosIng inventory). Consequently, natural compounds isolated and/or produced using biotechnological tools from microorganisms are already used for dermatologic purposes in topical cosmetic formulations. These products can aesthetically improve the skin’s appearance but can also prevent and/or treat age-related skin disorders. Beyond the “established” molecules, there are several small molecules and/or enzymes derived from microorganisms that have great potential to be used in cosmetics or cosmeceutical formulations (Table 1).

Interestingly, several biomolecules that are already included in the European Inventory of accepted cosmetic ingredients (CosIng inventory) [14] are registered for one of their biological activities, but are used differently in cosmetic applications. A characteristic example is kojic acid, which is registered as “antioxidant”, while the main application in cosmetics is its strong anti-tyrosinase activity, and thus its application as a skin whitening agent (Table 1).

Considering the immense microbial biodiversity and microbial adaptation to virtually any environment on earth, it is to be expected that microbes represent an extraordinary inventory of highly diverse structural scaffolds of biomolecules with potential skin protective activities. Although research on marine environment has started match later that the terrestrial environment, we have several cases where cosmetic applications and patents are in favor of marine-derived microorganisms. As mentioned in the case of MAAs known for their photo-protective activity, they are included in several patents for natural UV filters. However most of them were developed with microorganisms from marine environments (72.2%), while patents developed on terrestrial and fresh water microorganisms have not exceeded 21.4% and 2.4%, respectively [135]. This study reflects that to date, in some cases the terrestrial environment has been neglected.

Overall, taking into consideration that most of the world’s microbial terrestrial biodiversity remains largely uninvestigated and that microorganisms offer a sustainable, relatively low-cost and fast production process, we remain confident that in the near future, systematic research will reveal additional microorganisms that can be used as cell factories for producing high added value biomolecules with applications in the cosmetic industry as active ingredients.

## Figures and Tables

**Table 1:** Bioactive molecules produced by terrestrial microorganisms.

Classes of Natural Products			Bioactive Compound	Microorganism Classification (Kingdom; Species; Family)	Habitat	Biological Activity	CosIng Inventory^3^	References
Antioxidants^1^	Hydroxycinnamic acids		*p*-Coumaric acid	Fungi; *A. austroafricanus*; *Trichocomaceae*	Isolated from *Z. officinale* rhizome.	Antioxidant (DPPH, hydroxyl and nitric oxide radical-scavenging methods) Skin-whitening (inhibition of human tyrosinase and melanogenesis).		[31,32]
			Ferulic acid			Antioxidant (DPPH, hydroxyl and nitric oxide radical-scavenging methods) Photo-protective (SPFs: 1.3).	Antimicrobial	
			Cinnamic acid			Antioxidant (DPPH, hydroxyl and nitric oxide radical-scavenging methods).	Perfuming & skin conditioning agent	
			Chlorogenic acid	Cyanobacteria*;* *Dichothrix* sp*.; Rivulariaceae*		Antioxidant (DPPH radical scavenging activity, IC_50_: 6.41 μg/mL ABTS radical scavenging activity, IC_50_: 13.15 μg/mL Deoxyribose protective activity, IC_50_: 8.53 μg/mL)	Antioxidant, skin-conditioning & skin-protecting agent	[26]
			Caffeic acid	Cyanobacteria*; Aulosira fertilissima; Fortieaceae*		Antioxidant (DPPH radical scavenging activity, IC_50_: 6.34 μg/mL ABTS radical scavenging activity, IC_50_: 18.04 μg/mL Deoxyribose protective activity, IC_50_: 4.76 μg/mL)	Antioxidant & masking agent	
	Stilbenes		Resveratrol	Fungi*; Alternaria* sp.; *Pleosporaceae*	Isolated from grapes of *Vitis vinifera*	Antioxidant (inhibition of 8-OH-dG formation in DNA, IC_50_: 10.9) [33]^2^ Skin-whitening (inhibition of mushroom tyrosinase and of melanogenesis) [34]^2^ Preventive effect on lipid peroxidation [29]^2^	Antioxidant & skin protecting agent	[30]
				Fungi; *S. cerevisiae*; *Saccharomycetaceae*				[35]
				Bacteria; *E. coli*; *Enterobacteriaceae*				[36]
				Fungi; *S. cerevisiae; Saccharomycetaceae*	Isolated from Sugarcane.			[37]
				Bacteria; *E. coli*; *Enterobacteriaceae*	Obtained from a Genetic Stock Center, New Haven, CT.			[36]
				Bacteria; *Bacillus* sp.; *Bacillaceae*	Isolated from leaves of *Populus alba* L.			[38]
	Biphenyls		Altenusin	Fungi; *Botryosphaeria dothidea*; *Botryosphaeriaceae*	Collected from stems of white cedar (*Melia azedarach).*	Antioxidant (DPPH radical scavenging activity, IC_50_: 17.6 μM).		[39]
			5’, Methoxy-6- methylbiphenyl-3,4,3’-triol			Antioxidant (DPPH radical scavenging activity, IC_50_: 18.7 μM).		
	Naphthoquinone spiroketals		Palmarumycin C3	Fungi; *Berkleasmium* sp.; *Dematiaceae*	Isolated from healthy rhizomes of the medicinal plant *Dioscorea zingiberensis.*	Antioxidant (DPPH radical scavenging activity, IC_50_: 37.57 μM).		[40]
	Benzoic acids		Gallic acid	Cyanobacteria*; Limnothrix obliqueacuminata; Pseudanabaenaceae*		Antioxidant (DPPH radical scavenging activity, IC_50_: 3.53 μg/mL ABTS radical scavenging activity, IC_50_: 8.85 μg/mL Deoxyribose protective activity, IC_50_: 7.84 μg/mL)	Antioxidant	[26]
			Vanillic acid	Cyanobacteria; *Mastigocladus laminosus*; *Hapalosiphonaceae*		Antioxidant (DPPH radical scavenging activity, IC_50_: 416.7 μg/mL ABTS radical scavenging activity, IC_50_: 132.1 μg/mL Deoxyribose protective activity, IC_50_: 91.1 μg/mL)	Registered with no reported functions	
	Thiol peptides		Glutathione	Fungi; *P. pastoris*; *Saccharomycetaceae*		Antioxidant (minimizes lipid peroxidation in cellular membranes and other such targets that is known to occur with oxidative stress) [41]^2^ Skin-whitening (tyrosinase inhibitor) [42]^2^	Reducing agent	[43]
				Bacteria; *E. coli*; *Enterobacteriaceae*				[44]
	Carotenoids		Astaxanthin	Fungi*; X. dendrorhous; Filobasidiaceae*		Antioxidant (*in vitro* protection of biological membranes by an antioxidant mechanism) [45]^2^ Skin-whitening (inhibition of pigmentation, inhibition of melanin-generation)	Skin conditioning agent	[46]
				Chlorophyta; *H. pluvialis*; *Haematococcaceae*	Obtained from Algal Culture Center, Plant Physiology Institute, University of Gottingen, Germany.			[47]
			*β*-carotene	Bacteria; *E. coli; Enterobacteriaceae*		Antioxidant (αTEAC, FRAP and CL assay) [48]^2^	Skin conditioning agent	[1]
			Lycopene				Antioxidant	[49]
			Canthaxanthin	Bacteria; *Brevibacterium*; *Brevibacteriaceae*		Antioxidant (*in vitro* protection of biological membranes by an antioxidant mechanism) [45]^2^ Pigment	Cosmetic colorant	[50]
			Lutein	Chlorophyta; *Muriellopsis* sp.; *Chlamydomonadales incertae sedis*	Isolated from the Natural Park of the Empordá Marshes in Catalonia, Spain.	Antioxidant (Superoxide radical scavenging activity, IC_50_: 21 μg/mL Hydroxyl radical scavenging activity, IC_50_: 1.75 μg/mL Inhibition of lipid peroxidation: 2.2 μg/mL DPPH radical scavenging activity, IC_50_: 35 μg/mL ABTS radical scavenging activity: >100μg/mL Nitric oxide radical scavenging activity, IC_50_: 3.8 μg/mL) [51]^2^ Photo-protective (absorption of UVA rays)	Skin conditioning agent	[52]
			*Cis*-canthaxanthin	Actinobacteria; *D. maris*; *Dietziaceae*	Isolated from soil sample collected from the Kargil district of Jammu and Kashmir, India.	Antioxidant (inhibition of ROS generation in THP-1 cells, >80%)		[53]
	Polysaccharides (PSs)	Exopolysaccharides (EPSs)	EPS fraction (PS-I); rhamno galactan	Fungi; *F. solani*; *Nectriaceae*	Isolated from *A. scholaris.*	Antioxidant (scavenging potency, IC_50_: 578.54 μg/mL)		[54]
			Unknown EPS	Bacteria; *B. cereus*; *Bacillaceae*	Isolated from *A. annua* L.	Antioxidant: (DPPH radical scavenging activity, EC_50_: 3–5 mg/mL Superoxide radical scavenging activity, EC_50_: 2.6 mg/mL)		[55]
			Crude EPS	Bacteria*; P. polymyxa; Paenibacillaceae*	Isolated from *S. japonica* (Blume) Miquel.	Antioxidant (hydroxyl radical scavenging activity: 87.58% at 1 mg/mL)		[56,57]
			Mannose: fructose: glucose (2.6:29.8:1)			Antioxidant (hydroxyl radical scavenging activity: 76.73% at 1 mg/mL)		[56]
			Mannose: fructose: glucose (4.2:36.6:1)			Antioxidant (hydroxyl radical scavenging activity: 68.55% at 1 mg/mL)		
			Deproteinized EPS	Algae*; R. reticulata; Rhodellaceae*	Isolated from freshwater.	Antioxidant (superoxide anion radical scavenging activity: 328.48 U/L)		[58]
			Mannose: galactose (89.4:10.6)	Fungi; *Aspergillus* sp.; *Trichocomaceae*	Isolated from leaves of *Ipomoea pes-caprae* L.	Antioxidant	Mannose as humectant and galactose as skin-conditioning agent	[59]
			Rhamnose: glucose: glucuronic acid (2:2:1)	Bacteria*; B. tropica*; *Burkholderiaceae*	Isolated from Sugarcane.		Rhamnose as humectant & masking, glucose as humectant and glucuronic acid as humectant, chelating & buffering agent	[60]
			Unknown EPS	Chlorophyta*; Graesiella* sp.; *Chlamydomonadales incertae sedis*	Isolated from the hot spring ‘Ain Echffa’ (water temperature of 60 °C), Tunisia.			[61]
		Cell wall & exoskeleton PSs	Chitosan	Fungi; *R. oryzae*; *Mucoraceae*	Obtained from Culture Collection University of Gothenburg, Sweden.	Antimicrobial (higher activity on gram-positive bacteria, ex: Minimum inhibitory concentration (MIC) for *S. aureus*: 20 ppm) Moisturizing effect	Film forming & hair fixing agent	[62,63]
				Fungi; *R. japonicus*; *Mucoraceae*	Shanghai Institute of Industrial Microbiology, China.			
				Fungi; *M. indicus*; *Mucoraceae*	Obtained from Culture Collection University of Gothenburg, Sweden.			[64]
				Fungi; *A. niger; Trichocomaceae*	Isolated from the lichen *R. montagnei.*			[65]
			Chitin-glucan		-			[66]
Photo-Protective agents^1^	Melanins		-	Bacteria; *S. kathirae*; *Streptomycetaceae*	Isolated from soil samples.	Photo-protective (determination of SPF)	Skin protecting agent	[67]
				Bacteria; *Bacillus safensis; Bacillaceae*				[68]
	Indole derivatives		Violacein	Bacteria; *C. violaceum*; *Neisseriaceae*	Isolated from soil sample collected from the vicinity of an oil refinery wastewater treatment plant in Negeri Sembilan, Malaysia.	Photo-protective: broad absorption band extended out to 700 nm [69] Antibacterial (more efficient on Gram positive bacteria, ex: *S. aureus*, IC_50_: 6.99 μM)	Antimicrobial, antioxidant & cosmetic colorant	[70]
				Bacteria*; Duganella* sp*.; Oxalobacteraceae*.	Isolated from the glaciers of Tianshan, China.			[71]
			Scytonemin	Bacteria; *N. commune*; *Nostocaceae*	Collected from sandy soil in Ningbo University, China.	Photo-protective: UV absorbent (UV-A and UV-B region) [72] Antioxidant (dose-dependent DPPH scavenging activity of 12%, 33%, and 57% at concentrations of 0.5, 1.0, and 2.0 mg/mL, respectively. Ascorbic acid used as positive control)		[73]
			Streptochlorin	Bacteria; *S. roseolilacinus*; *Streptomycetaceae*	Isolated from soil.	Skin-whitening (anti-tyrosinase activity, IC_50_: 9 mM)		[74]
			Prodigiosin	Bacteria*; S. marcescens; Enterobacteriaceae*	Isolated from fields contaminated with pesticides.	Photo-protective Antibacterial (more efficient on Gram positive bacteria, ex: *S. aureus*, IC_50_: 0.68 μM)		[75]
				Bacteria; *Vibrio* sp.; *Vibrionaceae*	Isolated from estuarine waters of the Northern Adriatic Sea.			[76]
	Mycosporines		Palythine	Cyanobacteria*; Lyngbya* sp.; *Oscillatoriaceae*	Isolated from the bark of the rain tree *Albizia saman* (Jacq) Merr, Bangkok, Thailand.	Photo-protective (protection of HaCaT keratinocytes from solar-simulated radiation (SSR)-induced cell death), [77]^2^		[78]
			Asterina			Photo-protective		
			Unknown mycosporine-like amino acid			Antioxidant (DPPH scavenging activity of 14.5%, 53.0%, and 68.9% at 0.115, 0.230, and 0.460 mg/mL of MAAs, respectively. Ascorbic acid used as positive control)		
			Mycosporine-glutaminol-glucoside	Fungi*; R. minuta*; *Sporidiobolaceae*	Isolated from Patagonian natural environments	Photo-protective (UVB resistance of *X. dendrorhous* related to MGG production)		[79]
				Fungi*; R. slooffiae*; *Sporidiobolaceae*				
				Fungi*; R. lactosa*; *Sporidiobolaceae*				
				Fungi; *C. liquefaciens*; *Tremellaceae*	Isolated from a cold Arctic environment.			[80]
			Mycosporine–glutamicol–glucoside	Fungi; *C. cladosporioides*; *Cladosporiaceae*				
Skin-whitening agents^1^	Pyrones		Kojic acid	Fungi; *A. flavus*; *Trichocomaceae*	Isolated from *V. unguiculata.*	Skin-whitening (anti-tyrosinase activity, IC_50_: 0.014 mM)	Antioxidant	[81]
				Fungi; *A. oryzae*; *Trichocomaceae*	-			[82,83]
				Fungi; *A. parasiticus*; *Trichocomaceae*	Isolated from soil.			
				Fungi; *A. candidus*; *Trichocomaceae*	Isolated from soil.			
				Fungi; *A. flavus*; *Trichocomaceae*	Obtained from Department of Bioprocess Technology, University Putra, Malaysia.			
			(3R)-5-hydroxymellein	Endolichenic fungus	Isolated from the thalli of the lichen *Parmotrema austrosinense* (KoLRI no. 009806) collected from Jeju Island, Korea.	Photo-protective (damage recovery caused by UVB irradiation and inhibition of melanin synthesis) Antioxidant (DPPH radical scavenging, IC_50_: 30.8)		[84]
	Phenolic lactones		Ellagic acid (get by fungal bioconversion of ellagitannins)	Fungi; *A. niger*; *Trichocomaceae*	Obtained from DIA/UAdeC collection (Universidad Autonoma de Coahuila, Mexico).	Antioxidant (ABTS radical scavenging activity at 20 μg/mL: 93.9%). Skin-whitening (inhibition of melanogenesis)	Skin-conditioning agent	[85]
	Carboxylic acids		Lactic acid	Fungi; *R. oryzae*; *Mucoraceae*	Obtained from CBS-Centraalbureau voor Schimmelcultures, Utrecht, The Netherlands.	Skin-whitening [86]^2^ pH adjuster Exfoliant	Humectant, buffering & skin-conditioning agent	[87]
			Poly γ-glutamic acid	Bacteria; *Bacillus* sp.; *Bacillaceae*	Isolated from a soil sample collected at the Sugimoto campus of Osaka City University, Japan.	Skin-whitening Moisturizing (water-holding capacity: 56.9%) Antibacterial (more efficient on Gram-positive bacteria)		[88]
			Azelaic acid	Fungi; *Malassezia furfur*; *Malasseziaceae*		Skin-whitening (competitive inhibitor of tyrosinase: KI azelaic acid: 2.73x10^-3^ M) [89]^2^ Anti-bacterial and anti-acne effect Treatment of rosacea	Buffering & masking agent	[90,91]
	Tocopherols		Novel vitamine E succinate (bioconversion of vitamin E by modified *Candida antarctica* lipase B)	Fungi; *C. antarctica*; *Saccharomycetacea*		Skin-whitening effect		[92]
	Teichoic acids		Lipoteichoic acid	Bacteria; *L. fermenti; Lactobacillaceae*	Obtained from the National Collection of Type Cultures, Colindale, London.	Skin-whitening (inhibition of the intracellular activity of tyrosinase to 57.6% and 44.6% at 10 and 100 µg/mL of lipoteichoic acid)		[93]
	Quinones		Arbutin undecylenic acid ester	Bacteria; *B. subtilis*; *Bacillaceae*		Skin-whitening (anti-tyrosinase activity, IC_50_: 4.10^-4^ M)		[94]
Additives and other active ingredients^1^			Purpurogenone	Fungi; *P. purpurogenum; Trichocomaceae*		Pigment		[95]
			Unknown Anthraquinone derivative	Fungi; *P. oxalicum* var. Armeniaca*; Trichocomaceae*	Obtained from soil.			[96]
	Peptides & amino acid derivatives		Ectoine	Bacteria; *C. glutamicum; Corynebacteriaceae*		Photo-protective (*in vitro* inhibition of UVA- induced signal transduction in human keratinocytes as well as inhibition of UVA-induced formation of mitochondrial DNA mutations in human dermal fibroblasts), [97]^2^ Moisturizing effect	Skin conditioning agent	[98,99]
			Phenylalanine	Bacteria; *E. coli*; *Enterobacteriaceae*	New England Biolabs (NEB, Ipswich, MA).	Hair and skin conditioning effect		[100]
	Azaphilones		Mitorubrin	Fungi; *P. purpurogenum; Trichocomaceae*		Pigment		[95]
	Aromatic Aldehydes & alcohols		Benzaldehyde	Bacteria; *E. coli*; *Enterobacteriaceae*	New England Biolabs (NEB, Ipswich, MA).	Flavor, perfume (almond flavor)	Denaturant, flavoring, masking & perfuming agent	[100]
			Benzyl alcohol	Bacteria; *E. coli*; *Enterobacteriaceae*		Flavor, perfume Preservative Bacteriostatic	Perfuming, preservative, solvent & viscosity controlling agent	
			Vanillin	Bacteria; *E. coli*; *Enterobacteriaceae*		Flavor, perfume (vanilla flavor)	Masking agent	[101]
				Bacteria; *E. coli*; *Enterobacteriaceae*				[102]
				Bacteria; *B. fusiformis; Bacillaceae*	Isolated from soil.			[103]
			2-phenylethanol	Fungi; *A. gossypiii*; *Saccharomycetaceae*		Flavor, perfume (rose flavor) Preservative		[104]
				Fungi; *K. marxianus*; *Saccharomycetaceae*				[105]
	Terpenes		Limonene	Bacteria; *E. coli*; *Enterobacteriaceae*		Flavor, perfume (sweet citrus odor)	Deodorant, perfuming & solvent	[106]
	Glycolipids		Rhamnolipid	Bacteria; *P. aeruginosa; Pseudomonadaceae*		Moisturizing and surfactant	Emollient, emulsifying & stabilizing agent	[107]
			2,3,4,2’-trehalose tetraester	Bacteria; *R. erythropolis; Nocardiaceae*	Isolated from soil.	Surfactant		[108]

^1^ Compounds were grouped into four categories as described in the main text. Additional activities or common uses are reported. ^2^ Independent studies evaluating the specific compound. ^3^ EU Cosmetic ingredient database [14].

## Abbreviations

ABTS	2,2′-Azino-bis-3-ethylbenzthiazolin-6-sulphonic acid
CALB	*Candida antarctica* lipase B
CAGR:	compound annual growth rate
CDW	cell dry weight
DPPH	2,2-Diphenyl-1-picrylhydrazyl
EPSs	exopolysaccharides
HQ	hydroquinone
LTA	lipoteichoic acid
MAAs	mycosporine-like amino acids
MIC	minimum inhibitory concentration
γ-PGA	poly- γ-glutamic acid
RNS	reactive nitrogen species
ROS	reactive oxygen species
SPFs	sunscreen protection factors
SSR	solar-simulated radiation
SOD	superoxide dismutases
UVA	ultraviolet A
UVB	ultraviolet B
